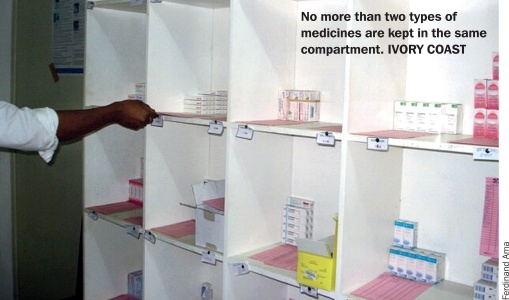# A simple paper-based system for stock management

**Published:** 2011-12

**Authors:** Ferdinand Ama, Paddy Ricard

**Affiliations:** Ophthalmologist, senior physician, Methodist Eye Centre, BP 81 Dabou, Ivory Coast. Email: amaferdinand@yahoo.fr; Consultant editor for the *Revue de Santé Oculaire Communautaire*, London, UK

The best systems create little extra work for staff and can be implemented almost without thinking in the course of a busy day. The system used in the stock room of our eye clinic in Ivory Coast illustrates this well.

All medicines, instruments, and consumables are held in the stock room. Each item has a specific code, which is displayed near that item on the shelf and is also kept on our accountant's computer.

Stock levels are monitored both by our accountant and by the person in charge of the stock room.

The accountant, who is in charge of ordering stock throughout the year, keeps a record on the computer of the stock levels of each item. Ordering is based on current stock levels and on our centre's statistics (e.g., there has to be enough consumables for our average of 20 cataract operations a week).

To keep accurate records on the computer, the accountant relies on the up-to-date records kept by the person in charge of the stock room. This is done by means of a simple system of individual stock cards.

A pink card is placed on the shelf next to each item. An open, compartmentalised shelving system — in which no more than two types of medication are held in the same compartment - ensures that products and stock cards are easy to find.

The stock cards are divided into two columns. The left-hand column states what was brought in or taken out, as well as the date. The right-hand column shows the number of items now in stock (the current stock level).

For example, when the person in charge of the stock room takes out two bottles of eye drops, she adds a new line to the card. In the left-hand column, she writes the date and that she has taken out two bottles of eye drops. She then updates the number in the right-hand column: the previous total minus two. Similarly, if twenty new bottles are brought in, she adds another new line and updates the number in the right-hand column: previous total plus twenty.

**‘Ordering is based on current stock levels and on our centre's statistics’**

The person in charge uses this system in all circumstances: when a patient buys medication, or when the centre's head nurse comes to collect consumables for the week's work.

The accountant can quickly check each item's stock level by glancing at the relevant card in the stock room. Once a week, stock cards are collected and the data entered into the computer.

This system of placing a single stock card right next to an item in the stock room can also be used when there is no member of staff specifically in charge of distributing stock, provided that everyone who is permitted to take items out of the stock room updates the relevant stock card.

**Figure:**